# Regulators of Actin Dynamics in Gastrointestinal Tract Tumors

**DOI:** 10.1155/2015/930157

**Published:** 2015-08-04

**Authors:** Konrad Steinestel, Eva Wardelmann, Wolfgang Hartmann, Inga Grünewald

**Affiliations:** Gerhard Domagk Institute of Pathology, University Hospital Münster, 48149 Münster, Germany

## Abstract

Reorganization of the actin cytoskeleton underlies cell migration in a wide variety of physiological and pathological processes, such as embryonic development, wound healing, and tumor cell invasion. It has been shown that actin assembly and disassembly are precisely regulated by intracellular signaling cascades that respond to changes in the cell microenvironment, ligand binding to surface receptors, or oncogenic transformation of the cell. Actin-nucleating and actin-depolymerizing (ANFs/ADFs) and nucleation-promoting factors (NPFs) regulate cytoskeletal dynamics at the leading edge of migrating cells, thereby modulating cell shape; these proteins facilitate cellular movement and mediate degradation of the surrounding extracellular matrix by secretion of lytic proteases, thus eliminating barriers for tumor cell invasion. Accordingly, expression and activity of these actin-binding proteins have been linked to enhanced metastasis and poor prognosis in a variety of malignancies. In this review, we will summarize what is known about expression patterns and the functional role of actin regulators in gastrointestinal tumors and evaluate first pharmacological approaches to prevent invasion and metastatic dissemination of malignant cells.

## 1. Introduction: Actin Dynamics in Invasion and Metastasis

**Figure 1:**
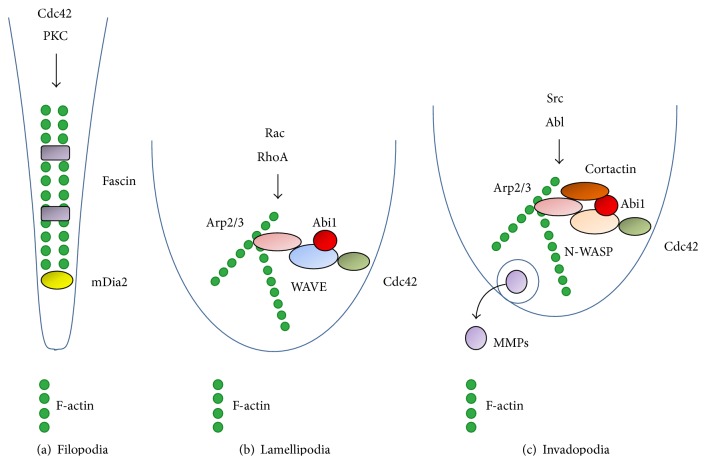
Simplified and schematic display of specialized cellular protrusions and key protein regulators involved in their formation and maintenance. (a) Filopodia consist of parallel actin filaments, which are formed by mDia2 upon Cdc42/protein kinase C signaling activity and then bundled by Fascin. (b) Rac/RhoA and/or Cdc42 signaling activates the WAVE complex, which, together with coregulators such as Abi1, stimulates Arp2/3-mediated actin branching and lamellipodial protrusion. (c) Invadopodia formation is mediated via N-WASP-mediated activation of Arp2/3 downstream Cdc42 signaling and might be further modulated by intracellular tyrosine kinase activity (Src, Abl). Furthermore, invadopodia secrete matrix metalloproteinases (MMPs) to degrade the extracellular matrix as a prerequisite for migration and invasion.

Reorganization of the actin cytoskeleton describes a process where cells actively alter the architecture of actin filaments to adjust cell shape in response to environmental requirements. Globular- (G-) actin is a highly conserved, polar protein with a molecular weight of 42 kDa that forms dimers and trimers in a process called actin nucleation; these structures then assemble to a double-stranded helical filament (F-actin) with a diameter of 7–9 nm (actin polymerization) [1–6]. Under baseline conditions, nucleation and polymerization are thermodynamically regulated processes whose kinetics depend on the amounts of available actin monomers, adenosine triphosphate (ATP), and divalent cations like calcium or magnesium ions [1, 7]. It has been shown that, under ideal conditions, up to 3000 subunits per second may be added to the growing actin filament, resulting in an elongation of 10 *μ*m in less than 2 s; however, spontaneous actin assembly is kinetically unfavorable for the cell [8, 9]. To allow for the establishment of a more complex architecture, actin filaments associate with different actin-associated proteins, such as actin-nucleating factors (ANFs) or nucleation-promoting factors (NPFs) that inhibit uncontrolled actin polymerization and support directed assembly of complex actin structures at distinct sites of the cell [10]. These structures then provide the cytoskeletal backbone for specialized cellular protrusions with well-defined functions in tumor cell invasion, such as filopodia, lamellipodia, and invadopodia (Figure 1). Filopodia are thin, hair-like cellular protrusions that consist of parallel actin bundles cross-linked by protein interaction partners such as Fascin, *α*-Actinin, Fimbrin, and Formins [11]. Filopodia sense changes in the cellular microenvironment, for example, growth factor concentrations, to guide the cellular movement through the surrounding matrix [4–6]. The cellular outgrowth of filopodia seems to precede lamellipodia formation when cells are plated on fibronectin; initial long filopodia are followed by protrusion of broad-based lamellipodia, a process that has been shown to depend on protein kinase C- (PKC-) mediated Fascin phosphorylation and inactivation [12]. Lamellipodia are broad-based cellular protrusions that expand their branched actin cytoskeleton into the direction of movement, which allows actin-myosin interactions to pull the cell through the surrounding tissue and thus follow the initial protrusion [13, 14]. The lamellipodial actin network is started by binding of the actin-related protein (Arp2/3) complex to the sides of preexisting actin filaments, allowing for actin branching in a characteristic 70° angle [10, 15]. The Arp2/3 complex is itself under tight control by nucleation-promoting factors (NPFs). Most of these proteins combine an actin-binding Wiskott-Aldrich syndrome (WAS) or verprolin homology 2 motif with a connector or Cofilin-binding region and an Arp2/3-complex-binding acidic peptide, collectively forming the so-called WCA or VCA domain [9, 16]. Their function is to bring actin filaments and the Arp2/3 complex in close proximity, thus facilitating actin polymerization. VCA-containing proteins, such as WASP family verprolin-homologous proteins (WAVE; also known as SCAR, three isoforms: WAVE 1–3), Wiskott-Aldrich syndrome proteins (WASP), neural WASP protein (N-WASP), WASP and SCAR homologue protein (WASH), and junction-mediating and regulatory protein (JMY), are designated class I NPFs. Cortactin, a class II NPF, does not contain a VCA domain; still, it has been shown to be capable of both activating the Arp2/3 complex and stabilizing newly generated filaments [17]. The actin structures that form invadopodia, the third type of specialized cellular protrusions, are closely related to the ones observed in lamellipodia and so are the signaling pathways that are responsible for their initiation and maintenance [13]. However, unlike lamellipodia, invadopodia have the ability to lyse extracellular matrix, for example, when invasive cancer cells migrate across the basement membrane, through collagen-rich tissue or towards blood or lymphatic vessels [18, 19]. Invadopodia will only form upon contact with a matrix substratum, and it has been shown that fibronectin-integrin crosstalk activates intracellular tyrosine kinase signaling, for example, via PKC, focal adhesion kinase (FAK), or Src kinase [20]. Accordingly, many Src substrates are enriched in invadopodia, such as the class I NPFs WASP/N-WASP or the class II NPF Cortactin [13, 21].

In the following section, we will summarize what is known about expression patterns and the functional role of individual actin-related proteins and nucleation-promoting factors in epithelial and nonepithelial tumors of the gastrointestinal tract.

## 2. Actin-Related Proteins and Nucleation-Promoting Factors in Gastrointestinal Tumors

### 2.1. Arp2/3

In the 220 kDa Arp2/3 complex, actin-related proteins 2 and 3 assemble with five other subunits (Arpc1–5) to bring the catalytic protein domain near the bound actin filament, thus contributing the first subunits to the new filament in an ATP-dependent process [9, 10, 15]. In detail, Arpc2 and Arpc4 bind to the parental filament, while Arp2 and Arp3 bind to the nascent daughter filament [22]. Activity of the Arp2/3 complex requires phosphorylation of tyrosine and threonine residues within Arp2 [23].

In gastrointestinal tumors, there have been conflicting observations about the role of Arp2/3 complex members. While overexpression of Arp2 and Arp3 has been reported during the progression from gastritis via intestinal metaplasia to cancer, Arp2 expression was strongest in intestinal metaplasia compared to gastritis and invasive carcinoma [24]. The authors did not assess Arp2/3 staining in healthy gastric mucosa. However, in that same study, Arp2 expression levels in tumor samples correlated with tumor size, depth of invasion, presence of venous invasion, and Cortactin expression but had no prognostic value for patient survival in multivariate analysis. Studies from another group, however, showed decreased gene expression of Arp2/3 complex members in gastric adenocarcinoma (GAC) [25, 26]. While CpG island methylation in the promoter of human Arp2/3 complex subunit p41-Arc (*ARPC1A*) was found in only 1 of 22 gastric carcinomas in the 2002 study [25], all 32 primary cancer samples examined for the 2004 study showed decreased expression of one or more Arp2/3 subunits. Moreover, 25/32 samples showed simultaneous downregulation of four or more subunits [26]. A possible explanation for these conflicting observations might be a confounding role for inflammation-induced carcinogenesis, since accumulation of Arp3 in gastric epithelial cells infected with* H. pylori* has been previously reported [27]. However, the impact of* H. pylori *on cytoskeletal rearrangement in gastric epithelial cells has been shown to be independent of Cdc42, Rac1, and Arp2/3 in another study [28].

In colorectal cancer (CRC), increased expression of Arp2 and Arp3 in tumor cells and surrounding stroma can be observed during colorectal carcinogenesis via the adenoma-carcinoma sequence and is strongest in invasive carcinoma [29]. Moreover, high expression levels of Arp2 mRNA in CRC tissue have been associated with liver metastasis [30].

*ARPC1A*, the gene encoding for the p41 subunit of Arp2/3, is frequently overexpressed in pancreatic cancer due to 7q21-q22 gene amplification, and RNA interference experiments show a significant decrease in tumor cell migration and invasion upon* ARPC1A *and* ARPC4 *knockdown [31, 32]. Taken together, the data on Arp2/3 complex and its individual subunits in gastrointestinal tumorigenesis, tumor progression, and formation of metastasis is conflicting; the expression changes of Arp2/3 and other NPFs in various GI tumors are summarized in Table 1. A general statement on the role of the protein complex in cancer development and progression might as yet be impossible. This can be due to the variety of intracellular signaling cascades targeting individual subunits and protein regulators of Arp2/3 [33]. More detailed analyses on the tumorigenic and promigratory impact of different Arp2/3 complex subunits would therefore contribute to a more detailed understanding of the biology of this multifaceted regulatory network.

### 2.2. Fascin

Fascin is a highly conserved actin-bundling protein with three isoforms: while Fascin-1 is nearly ubiquitously expressed during embryogenesis, its expression is later restricted to endothelium, neuronal tissue, and testis [11]. Fascin-2 and Fascin-3, respectively, are expressed in retinal epithelium and testis only [34]. Fascin is phosphorylated by protein kinase C (PKC), which regulates its actin-bundling activity dependent on current microenvironmental conditions which are communicated via surface integrins [11]; however, increased expression levels of Fascin have been described in several gastrointestinal malignancies as well as in invasive breast cancer and malignant melanoma. In esophageal squamous cell carcinoma, overexpression of Fascin is associated with tumour spread, lymph node metastasis, and poor prognosis, and downregulation of the protein diminished invasive properties* in vitro* [35]. In gastric cancer, Fascin is upregulated in adenomas and carcinomas compared to adjacent nonneoplastic mucosa [36]. A significant proportion of colorectal adenomas show focal Fascin expression, and positive Fascin immunohistochemistry is associated with shorter survival in CRC stage III/IV patients [37]. Similar results have been reported for pancreatic malignancies, where Fascin is overexpressed in intraductal papillary mucinous neoplasms, and expression correlates with histologic tumor grade [38]. Finally, Fascin-positive hepatocellular carcinomas more frequently display portal venous invasion, bile duct invasion, intrahepatic metastasis, and poor clinical outcome compared to Fascin-negative tumors [39].

### 2.3. N-WASP

Wiskott-Aldrich syndrome protein (WASP), which is expressed only in hematopoietic cells, and its ubiquitously expressed analogue N-WASP are class I NPFs that enhance Arp2/3-mediated actin polymerization upon stimulation by Cdc42 and/or phosphatidylinositol-4,5-bisphosphate (PIP2) [40]. Wiskott-Aldrich syndrome is caused by mutations in the WASP-encoding* WAS* gene and characterized by microthrombocytopenia, eczema, recurrent infections, and autoimmunity due to dysfunctional reorganization of the actin cytoskeleton [41]. In esophageal squamous cell carcinoma, N-WASP is expressed in the cytoplasm of tumor cells, and protein expression correlates with lymph node metastasis and pathological stage; however, mRNA expression is not significantly different between cancer and noncancerous tissue, and N-WASP protein expression is not a prognostic factor for survival in Kaplan-Meier analysis [42]. Although it has been shown that cytoskeletal alterations play a central role in the response of gastric epithelial cells to CagA, the major virulence factor of* H. pylori *[43], the role of the protein in the progression of gastric adenocarcinoma has so far not been thoroughly studied. In colorectal cancer, one study reported an inverse correlation between N-WASP expression and tumor aggressiveness and postulated a possible role for N-WASP as a tumor suppressor protein via the focal adhesion kinase (FAK) pathway; however, the authors did not specify the method of quantification of immunostaining in that study, making the results difficult to interpret [44]. N-WASP was among the upregulated genes in liver metastases of CRC compared to primary tumors in another study, concordant with the central role of N-WASP in invadopodia formation and maintenance [45]. Therefore, the exact role of N-WASP in CRC is yet unclear. In pancreatic ductal adenocarcinoma, N-WASP overexpression is associated with perineural invasion and unfavorable prognosis [46], while the protein is overexpressed compared to healthy liver tissue and an independent prognostic factor for overall survival in HCC [47].

### 2.4. WAVE

WAVE/SCAR, in contrast to WASP, is itself part of a tightly regulated multiprotein complex containing PIR121, Nap1, HSPC300, and Abi1; the complex is regarded as a “signaling hub” to integrate and process intracellular signaling pathways towards activation or inhibition of actin nucleation and branching [48, 49]. It has been shown that removal of Abi1 or any other subunit of the WAVE complex impairs its activity and leads to rapid degradation of WAVE, underlining the importance of structural integrity of the complex [50–52]. Contrary to the postulated promigratory role of other actin-related proteins and NPFs, WAVE2 has been shown to be downregulated in gastric carcinoma, and, surprisingly, WAVE2 knockdown increased gastric carcinoma cell growth, invasiveness, motility, and adhesiveness and suppressed epithelial-mesenchymal transition* in vitro* [53]. Conversely, it has been reported that miRNA146a-induced downregulation of WAVE2 results in impaired migration and invasion, and WAVE3 expression enhances metastasis, invasion, and proliferation activity via upregulation of Snail in gastric cancer cell lines [54, 55]. WAVE2 is strongly expressed in colorectal carcinoma cells, but only weakly in colonic epithelium maintaining normal structure; furthermore, colocalization of Arp2 and WAVE2 is associated with tumor cell budding, an established marker for aggressive tumor behavior in colorectal carcinoma and an independent risk factor for liver metastasis in CRC [30, 56]. In hepatocellular carcinoma, WAVE2 expression is correlated with the presence of multiple tumor nodules, absence of capsule formation, higher tumor grade, and venous invasion; moreover, it is an independent prognostic factor for poor prognosis [57]. Taken together, similar to the previously discussed NPFs, members of the WAVE protein complex can act as either suppressors or enhancers of tumor progression. This is not surprising, having in mind that WAVE2, for example, can act as a tumor suppressor in benign tumors (stabilizing cell-cell adhesion) and, at the same time, as a driver of epithelial-mesenchymal transition in migrating cancer cells (inducing actin meshwork formation at the cellular leading edge) [58]. Therefore, the role of WAVE complex proteins seems to depend on the respective tumor entity and its pathological stage, a fact that should be considered in the planning of future tissue-based studies.

### 2.5. Cortactin

Cortactin, a class II NPF, is a key regulator of invadopodia formation and maintenance [17, 59, 60]. It interacts with class I NPFs (e.g., N-WASP) and their respective interaction partners, such as WIP, and colocalizes with Abi1 at sites of matrix degradation by MDA-MB-231 cells [61–63]. Accordingly, presence of Cortactin has been shown to be crucial for migratory behavior of both neoplastic and nonneoplastic cells [64, 65]. Accordingly, the* CTTN* gene encoding for Cortactin is located in the 11q13 region, a chromosomal region that is frequently amplified in malignant tumors [66].

Cortactin is overexpressed in premalignant lesions, early stage dysplasia, and carcinoma* in situ* of the esophagus; together with Fascin and Survivin, high protein expression levels of Cortactin have been shown to be associated with worse prognosis of patients with ESCC [67, 68]. In another study, amplification and overexpression of the* CTTN* gene correlated with lymph node metastasis in ESCC, while* in vitro* analyses showed a role for Cortactin in anoikis resistance, tumor growth, and lung metastasis of ESCC cells via the PI3K/Akt pathway [69]. Finally, a tumor-promoting role for Calreticulin has been shown to be linked to dysregulation of Cortactin expression, and vascular endothelial growth factor C (VEGF-C) contributes to tumor growth and metastasis via Src-miR326-mediated overexpression of Cortactin in ESCC [70, 71]. In GAC, ambivalent results have been reported about a possible prognostic value of Cortactin expression levels, depending on which antibody was used for immunohistochemical analyses, which might be due to the fact that the migratory-promoting function of Cortactin depends on phosphorylation of the protein on serine residues S405 and S418 [36, 72]. However, Cortactin expression showed an association with poor tumor differentiation, more advanced tumor, lymph node and metastasis (TNM) stage, tumor recurrence, and poor survival in GAC in other studies [73, 74]. In* in vitro* studies and a mouse tumor model, stable overexpression of Cortactin promoted while downregulation of the protein inhibited SGC-7901 gastric cancer cell migration, invasion, and metastatic spread [75]. In CRC, irrespective of individual histological differentiation grade, Cortactin is overexpressed compared to normal colorectal epithelium, and immunostaining scores correlate with T and M stage of the tumor [76]. The dominant expression form in CRC seems to be the protein migrating at 85 kD in SDS-PAGE, which has been proposed to represent a phosphorylated form of the protein [76, 77]. Moreover, in the 2009 study by Lee et al., strong immunopositivity for Cortactin was detected only in a minority of primary tumors but, in most metastases, supporting a role for the protein in the gain of a promigratory tumor cell phenotype in CRC [76]. These findings were supported by results from other authors showing that Cortactin expression correlated with tumor invasion, histological grade, and preoperative CEA level; moreover, it was an independent prognostic factor for both disease-free and overall survival in stages II-III CRC [78, 79]. However, since Cortactin is almost ubiquitously expressed, subcellular localization of the protein in tumor cells seems to be critical for its role in cell migration and metastasis. Accordingly, one study reported interaction of Cortactin and zonula occludens-1 (ZO-1) in migrating or polarized colorectal carcinoma cells [80], and we previously showed localization of the protein to sites of matrix degradation in a gelatin-based extracellular matrix degradation assay* in vitro *[81]. In hepatocellular carcinoma, Cortactin is overexpressed compared to healthy liver tissue, and protein expression correlates with histological differentiation, metastasis, T stage, and poor prognosis; moreover, an increase in HCC cell migration and metastasis has been observed upon Cortactin overexpression* in vitro* [82, 83]. Another study demonstrated a significant correlation between Cortactin expression and liver capsule integrity, portal vein cancer embolization, TNM stage, and distant metastasis in HCC [84]. The promigratory function of Cortactin in HCC might be linked to Src-mediated phosphorylation and activation of the protein [85].

In adenocarcinomas of the pancreas and ampulla of Vater, overexpression of Cortactin (and Fascin-1) is associated with poor tumor differentiation, advanced tumor stage, and shorter survival [86]. Pancreatic cancer cell invasion has been shown to be dependent on Ezrin-Cortactin-driven formation of podosomes, specialized cellular protrusions with a function similar to invadopodia [20, 87].

### 2.6. Abi1–3

Abelson interactor 1 (Abi1), a substrate for the eponymous Abelson nonreceptor tyrosine kinase (Abl) and one of the protein components of the WAVE complex, has itself been shown to regulate the activation of Rac downstream tyrosine kinase receptor signaling, thus modulating the response of the WAVE complex in a feedback loop [88, 89]. It has further been shown that removal of Abi1 or any other subunit of the WAVE complex impairs its activity and leads to rapid degradation of WAVE [50–52], underlining the importance of the structural integrity of the complex. However, Abi1 does not only regulate WAVE, but also cooperates with Cdc42 to enhance N-WASP activity; moreover, in the absence of WAVE, Abi1 enters a complex with Formins of the Diaphanous family, which are regulators of actin turnover at the barbed end of the actin filament [90, 91]. These multiple tasks and potential interaction partners make Abi1 one of the key regulating proteins during dynamic reorganization of the actin cytoskeleton, and overexpression of the protein in breast and ovarian cancer has been reported [92, 93]. Like its homologue Abi1, Abi2 is part of the WAVE complex and promotes Abl-mediated phosphorylation of WAVE2, while Abi3/NESH, although sharing a similar protein domain structure and although being part of the WAVE complex, does not seem to have a similar effect [94].

Despite its central role in lamellipodia formation and cell migration, one study reported downregulation of Abi1 expression in gastrointestinal tract tumors (ESCC and esophageal adenocarcinoma, GAC, and CRC) [95]. Currently, data on Abi proteins in upper GI tract tumors is sparse, making it difficult to sum up their relevance. Our own group investigated the role of Abi1 in CRC and showed upregulation of Abi1 during the adenoma-carcinoma sequence of colorectal carcinogenesis as well as in* KRAS*-mutant hyperplastic polyps with constitutive activation of EGFR signaling pathway [96]. Consistent with that, another group showed upregulation of the Abi2 gene together with the genes for the fibroblast growth factor, profilin-2, and radixin during tumorigenesis of inflammatory bowel disease- (IBD-) associated CRC [97]. In invasive CRC, we could show that expression and Y435 phosphorylation of Abi1 are associated with an aggressive tumor phenotype and promote tumor cell adhesion, extracellular matrix degradation, and invasion by CRC cells [81]. Accordingly, we demonstrated that application of STI571/Glivec inhibited Abi1 tyrosine phosphorylation, extracellular matrix degradation, and tumor cell invasion in that study [81]. First results from a UK-based research group show that Abi1 and its interaction partners Eps8 and Sos1 are overexpressed in PDAC, and knockdown of one of the proteins impairs *α*v*β*6 integrin-dependent pancreatic cancer cell invasion* in vitro *[98, 99]. However, these results have to be regarded as preliminary and still require final confirmation.

### 2.7. Cofilin

Contrary to most of the previously mentioned regulators of actin dynamics, Cofilin has mainly a contrary function as an actin-depolymerizing factor (ADF). As such, together with actin-nucleation and nucleation-promoting factors, it contributes to a tight temporal and spatial regulation of actin reorganization [100]; however, as has been pointed out before, it acts via severing and fragmentation of actin filaments rather than really affecting the rate of actin depolymerization [1]. Interestingly, since Cofilin-mediated fragmentation provides the Arp2/3 complex with new filaments for dendritic nucleation, it may even amplify the function of ANFs under some circumstances [101]. Taken together, a tight balance between Cofilin activation and inhibition is crucial for directed movement of the cell. In malignant tumors, overall activation of the Cofilin pathway has been described in breast cancer, while overexpression of the protein decreased the invasive potential of lung cancer cells [102, 103].

Cofilin has been reported to be overexpressed in ESCC, a finding that could since then be confirmed by others [42, 104]. However, although the protein was included in a test cohort of 110 ESCC surgical specimens in another study, it was not part of the three-protein signature model with high prognostic value that could be confirmed by the authors in an independent validation cohort [105].

Expression of Cofilin in BGC-823 gastric cancer cells has been described, but data on the correlation between expression levels of the protein and clinicopathological features are missing [106]. This would be of great interest, since one study described that application of LCH-7749944, an inhibitor of p21-activated kinase 4, inhibits migration and invasion of gastric cancer cells through inhibition of the PAK4/LIMK1/Cofilin signaling cascade [107]. A more detailed review on possible pharmacological interference with Cofilin is given below.

In colon cancer cells that show highly invasive properties compared to weakly invasive ones, Cofilin is overexpressed and colocalizes with actin filaments in the cell periphery [108]. This is supported by data showing that overexpression of the protein enhances cell spreading and Cofilin/F-actin colocalization in LS180 CRC cells [109]. Diallyl disulfide- (DADS-) mediated downregulation of LIM kinase-1 (LIMK1) suppresses LW480 CRC cell migration by impairing activity along the Rac1-ROCK1/PAK1-LIMK1-ADF/Cofilin signaling pathway [110]. Finally, it has been shown that the frequently observed overexpression of CD44 in CRC is mechanistically linked to Cofilin expression [111]. Despite these experimental observations, to the best of our knowledge, expression patterns of Cofilin in CRC as well as possible correlations with clinicopathological characteristics have so far not been investigated.

Cofilin is downregulated in highly metastatic hepatocellular carcinoma cells (MHCC97-H) compared to the weakly metastatic strain MHCC97-L [112], while, in pancreas, Cofilin is upregulated in cancerous versus noncancerous tissue [113].

## 3. Actin Dynamics in Nonepithelial and Infrequent Epithelial Tumors

Although gastrointestinal stromal tumors (GISTs) frequently show dysregulation of Src and Abl kinase signaling, the expression and function of the actin-related proteins downstream these signaling cascades have not been investigated so far in this tumor entity [114]. However, this would be of clinical interest, since application of tyrosine kinase inhibitors (TKIs) has been shown to attenuate Kit/Cortactin interaction in mast cell leukemia [115]. Moreover, the effect of STI571/Glivec treatment on phosphorylation and function of Abelson interacting proteins (Abi1/2) has also not been investigated in GIST. It might be conceivable that the use of TKIs for the treatment of GIST has in fact functional consequences on actin dynamics and the gain of an invasive phenotype in this tumor entity.

Finally, it has been shown that, in endocrine tumors of the gastrointestinal tract, Cofilin expression correlates with the extent of tumor invasion [116].

## 4. Actin Dynamics as Potential Therapeutic Targets

CK-0944636 and CK-0993548 are small molecule inhibitors of the Arp2/3 complex; while CK-0944636 binds between Arp2 and Arp3 and thus blocks their movement into the active conformation, CK-0993548 changes the conformation of Arp3 by insertion into its hydrophobic core [117]. However, antitumoral or anti-invasive properties of both compounds have so far not been evaluated. The guanine-binding domain of the class I NPFs WASP/NWASP bound and stabilized in the autoinhibited fold by wiskostatin, a cyclic peptide and carbazole derivative first published in 2004 [118]. It has been shown that application of the compound blocks the outgrowth of neuronal processes and impairs NK cell migration and spontaneous motility as well as chemotactic motion by* Dictyostelium discoideum *cells [119, 120]. Interestingly, surface expression and activity of cystic fibrosis transmembrane conductance regulator (CFTR) are also suppressed by application of wiskostatin, indicating a role for N-WASP in regulating CFTR expression and activity at the cell surface [121]. Our own results from rat hippocampal neurons and mouse neural stem cells support the idea that activity of N-WASP exerts an influence on membranous ion channel expression and activity, since, in our models, both N-WASP and the small conductance potassium activated calcium channel 3 (KCNN3, SK3) were indispensable for actin dynamics in early neurogenesis [119]. This is of special interest, since the key role of intracellular calcium hemostasis in the regulation of actin dynamics is widely recognized [122, 123]. In cancer cells, wiskostatin effectively blocks breast cancer cell migration in a concentration of 50 *μ*M; moreover, treatment with 5 and 10 *μ*M wiskostatin significantly reduced the matrix degradation area as well as the percentage of invadopodia in head and neck squamous carcinoma cells [124]. This finding might be explained by the key role of N-WASP in invadopodia formation and maintenance [13]. Another compound that stabilizes the overall autoinhibited confirmation of N-WASP is 187-1, which is a 14-amino acid cyclic peptide published by Peterson et al. for the first time in 2001 [125] (reviewed by Peterson himself in 2004 [126]). However, to the best of our knowledge, this compound has not been tested for its possible anti-invasive properties in* in vitro* or even* in vivo* models yet [127]. As mentioned above, we have previously shown that application of STI571/Glivec effectively inhibits CRC tumor cell invasion through the inhibition of Abi1 phosphorylation [81]. The actin-bundling activity of Fascin can be targeted by synthetic analogues to migrastatin, a natural secretion product of* Streptomyces*, which effectively inhibits tumor cell migration, invasion, and metastasis [128]. For Cofilin, it has been reported that angiogenesis inhibitors such as Endostatin, thrombospondin-1, fumagillin, endothelial monocyte activating polypeptide II, and TNP-470 alter the phosphorylation state of Cofilin and induce formation of stress fibers in endothelial cells, which might have an impact on tumor-induced neovascularization and blood supply [129]. In U937 histiocytic lymphoma cells, it has been shown that Herbimycin A inhibits or, at least, significantly reduces dephosphorylation and translocation of Cofilin to the cell periphery [130]. However, results on the effects of pharmacological compounds or inhibitors targeting Cofilin in gastrointestinal tumors have so far not been published.

## 5. Summary/Conclusions

Taken together, there is strong evidence for a central role of actin-binding proteins in tumor formation, tumor cell invasion, and metastatic spread of gastrointestinal tract malignancies (Table 1). Fascin expression seems to be quite consistently associated with tumor aggressiveness, while the results for Arp2/3 complex members are partly contradictory and also vary between different tumor localizations. Most findings support a tumor-promoting function for Cortactin that might be linked to the essential role of the protein in invadopodia formation and maintenance, but the results for class I NPFs (N-WASP/WAVE), Abi1/2, and Cofilin are conflicting, especially in upper gastrointestinal tract tumors. A possible explanation might be that these proteins are subject to a variety of intracellular signaling cascades that might exert both promigratory and antimigratory effects depending on current microenvironmental conditions, such as inflammatory response, oxygen supply, and oncogene mutation status of the tumor cell, since these confounding factors have not been standardized in most of the studies that have been summarized above. We have shown, for example, that* KRAS* mutation status and an inflammatory response have an impact on Abi1 expression levels in CRC cells [96]; taking this into account, the mere expression of actin-binding proteins does not necessarily imply a more aggressive tumor behavior, all the more since most of these proteins are subject to phosphorylation and dephosphorylation events with great impact on their respective activity. Similar results have been shown for WAVE complex members (WAVE2) [58]. Despite the strong* in vitro* evidence for the essential role of these proteins in tumor cell migration and invasion, their possible use as pharmacological targets has so far not been thoroughly investigated. Therefore, further studies should focus on the functional characterization of these proteins as potential targets for antitumoral and/or anti-invasive therapies in gastrointestinal tract tumors.

## Figures and Tables

**Table 1:** Expression patterns of actin-binding proteins in gastrointestinal tract tumors.

	Arp2/3	Fascin	N-WASP	WAVE	Cortactin	Abi1–3	Cofilin
ESCC	—	↑ [35]	↑ [42]	—	↑ [67–71]	↓ [95]	↑ [42]

GAC	↑ [24] ↓ [25, 26]	↑ [36]	—	↓ [53] ↑ [54]	↓ ↑ [36] ↑ [72–75]	↓ [95]	(↑) [106]

CRC	↑ [29, 30]	↑ [37]	↓ [44] ↑^*∗*^[45]	↑ [30]	↑ [76, 78–81]	↓ [95] ↑ [81, 96, 97]	(↑) [108–111]

HCC	—	↑ [39]	↑ [47]	↑ [57]	↑ [82–85]	—	↓ [112]

PDAC	↑ [31, 32]	↑ [38]	↑ [46]	—	↑ [86, 87]	↑ [98, 99]	↑ [113]

ESCC: esophageal squamous cell carcinoma; GAC: gastric adenocarcinoma; CRC: colorectal carcinoma; HCC: hepatocellular carcinoma; PDAC: pancreatic ductal adenocarcinoma.

^*∗*^In metastases versus primary tumors.
